# Using pre-training and interaction modeling for ancestry-specific disease prediction using multiomics data from the UK Biobank

**DOI:** 10.1371/journal.pone.0336861

**Published:** 2025-12-01

**Authors:** Thomas Le Menestrel, Erin Craig, Robert Tibshirani, Trevor Hastie, Manuel Rivas

**Affiliations:** 1 Institute for Computational and Mathematical Engineering (ICME), School of Engineering, Stanford University, Stanford, California, United States of America; 2 Department of Statistics, Stanford University, Stanford, California, United States of America; 3 Department of Biomedical Data Science, Stanford University, Stanford, California, United States of America; Calico: Calico Life Sciences LLC, UNITED STATES OF AMERICA

## Abstract

Recent genome-wide association studies (GWAS) have uncovered the genetic basis of complex traits, but show an under-representation of non-European descent individuals, underscoring a critical gap in genetic research. Prediction models trained primarily on European ancestry often fail to generalize to diverse populations, leading to reduced accuracy and potential health disparities. Here, we assess whether incorporating interaction modeling and pretraining into disease prediction models can improve performance. We evaluated the performance of Group-LASSO INTERaction-NET (glinternet) and pretrained lasso in disease prediction focusing on diverse ancestries in the UK Biobank. Models were trained on multiomic data from White British and other ancestries and validated in a cohort of more than 96,000 individuals for 8 diseases. Of the 96 trained models, we report 16 with statistically significant incremental predictive performance in terms of ROC-AUC scores (p-value<0.05), found for diabetes, arthritis, gall stones, cystitis, asthma, and osteoarthritis. Our findings suggest that interaction terms and pre-training can modestly improve prediction accuracy, but these effects are not consistent across all diseases. Our code is available at (https://github.com/rivas-lab/AncestryOmicsUKB).

## Introduction

Genome-wide association studies (GWAS) have significantly advanced our understanding of the genetic basis of complex traits and diseases [1,2]. Yet, these studies have predominantly focused on individuals of European descent, leading to under-representation of non-European ancestry in genomic databases [3,4], despite ancestry being a crucial factor in disease susceptibility [5]. This is also the case for the UK Biobank, which contains approximately 75% data from White British individuals and less than 10% from diverse ancestries such as Related (9.9%), Admixed (6.4%), Non-British European (5.6%), South Asian (1.8%), African (1.5%) and East Asian (0.4%) [6].

Efforts to address the challenge of ancestry-specific disease prediction have varied. Polygenic risk scores (PRS) have been trained for disease prediction using UK Biobank data [7,8]; however, their efficacy diminishes when applied to non-European individuals. PCA approaches have also been developed to create ancestry-specific PRS for disease prediction [9]. Alternative strategies employing LASSO variants and advanced machine learning techniques like AutoEncoders have been attempted [10,11]. Yet, these methods struggle with non-linearity and lack the interpretability of simpler models. LASSO and Cox proportional hazard models have been extensively used for this problem but under-perform when dealing with non-linearity as well as because of the proportional hazard assumption failing in practice [12–15]. Finally, how multiomics data beyond genetics improve disease risk prediction in non-European populations is unclear and most studies have focused on single data entity as well as single diseases [16–18].

Our study uses pre-training and interaction modeling across multiple ancestries and individual ancestries jointly to facilitate the application of patterns identified in White British data to other ancestries. Interaction modeling allows adjustement of coefficients of certain variables based on specific covariates, often age, sex or PRS for a given disease. This lets the model capture interactions between features, for instance, the effect of a genetic variant which might be different depending on the ancestry of a patient or environmental factors. On the other hand, pre-training on a large dataset with multiple ancestries is beneficial when underlying genetic patterns may be complex. After the initial broad training, the model is fine-tuned on a smaller, more specific dataset. This is crucial when dealing with PRS for diverse ancestries because it allows the model to adapt to the nuances of specific genetic backgrounds that may not be well represented in the larger dataset.

We compiled clinical data from over 96,000 individuals spanning 8 diseases with demographic, metabolomic, genetic and biomarker data, thus having a dataset with multiomic data and multiple diseases. We identified 8 common diseases to use for our study: diabetes (DIA), asthma (AST), chronic renal failure (CRF), osteoarthritis (OST), myocardial infarction (MI), cystistis (CYS), gall stones (GS) and arthritis (ART). We trained logistic regression, glinternet, and pretrained lasso models on datasets containing all ancestries (“All”) and White British plus a single ancestry (“Mix”) then predicted on ancestry-specific datasets for each ancestry. This approach improves prediction accuracy across a diverse range of ancestries and maintains the level of interpretability achieved in previous studies.

## Materials and methods

## Ethics statement

This research has been conducted using the UK Biobank Resource under Application Number 24983, “Generating effective therapeutic hypotheses from genomic and hospital linkage data” (http://www.ukbiobank.ac.uk/wp-content/uploads/2017/06/24983-Dr-Manuel-Rivas.pdf). Based on the information provided in Protocol 44532, the Stanford IRB has determined that the research does not involve human subjects as defined in 45 CFR 46.102(f) or 21 CFR 50.3 (g). All participants of the UK Biobank provided written informed consent (information available at https://www.ukbiobank.ac.uk/2018/02/gdpr/).

### L1-penalized logistic regression

The Lasso regression in the context of a binomial response variable can be expressed as an optimization problem. The objective is to minimize the cost function that comprises the log-likelihood of a binomial distribution along with an *L*_1_ penalty term. The mathematical formulation is given by:

$$\min_{\beta }\left(-\sum_{i=1}^{n}\left[y_{i}\log(p_{i})+(1-y_{i})\log(1-p_{i})\right]+\lambda \sum_{j=1}^{p}|\beta_{j}|\right)$$ (1)

where *β* represents the coefficients of the model, *n* is the number of observations, *y*_*i*_ is the response variable which can take values 0 or 1, *p*_*i*_ is the probability of *y*_*i*_ being 1, often modeled as $p_{i}=\frac{1}{1+e^{-\beta^{T}x_{i}}}$, where *x*_*i*_ is the vector of explanatory variables for the *i*-th observation and *λ* is the regularization parameter, controlling the strength of the *L*_1_ penalty term.

### Glinternet

Glinternet fits a first-order interaction model while enforcing a strong hierarchy: an interaction effect between two variables is considered in the model only if both main effects are also included [19]. Let $X=(X_{1},\ldots ,X_{p})$ denote the original variables and *Y* the response. This approach is effective in the context of high-dimensional data, where interactions between variables provide significant insights but also pose challenges in terms of model complexity and interpretability. The method proceeds as follows:

The group-lasso extends the standard lasso by allowing coefficients to be penalized in predefined groups rather than individually. Suppose the features are divided into *p* groups (potentially of different sizes), where *X*_*j*_ denotes the design matrix corresponding to group *j* and *Y* the response vector. The group-lasso estimates the coefficients ${\hat{\beta }}_{j}$ by solving


$$\min_{\mu ,\beta }\frac{1}{2}{\left\|Y-\mu 1-\sum_{j=1}^{p}X_{j}\beta_{j}\right\|}_{2}^{2}+\lambda \sum_{j=1}^{p}\gamma_{j}\|\beta_{j}{\|}_{2}.\;\left(10\right)$$

If each group contains only a single variable, this reduces to the standard lasso objective. In our application, each matrix *X*_*j*_ represents a group of features associated with one variable - including its main effect and all its interaction terms. The group-lasso thus encourages group-wise sparsity, setting entire coefficient vectors $\beta_{j}$ to zero when their joint contribution to predicting *Y* is weak. When ${\hat{\beta }}_{j}$ is nonzero, all of its components (main and interaction terms) are typically nonzero as well. In this way, *glinternet* combines the interpretability and regularization strength of the group-lasso with the flexibility of modeling pairwise (first-order) interactions.

**1. Candidate set construction.** Form a candidate set 𝒞 of features. If variable screening is desired, 𝒞 contains only selected main effects and their associated interactions; otherwise, 𝒞 includes all main effects and all pairwise interactions:


$$𝒞=\{X_{1},\ldots ,X_{p}\}\cup \{X_{i:j}:1\leq i<j\leq p\},$$

where *X*_*i*:*j*_ denotes the elementwise product of *X*_*i*_ and *X*_*j*_. Define a group for each variable:


$$G_{i}=\{\text{main effect }X_{i}\}\cup \{X_{i:j}:j>i\}.$$

Each group *G*_*i*_ contains the main effect of variable *i* and all interactions involving *i*.

**2. Group-lasso fitting.** Let $\beta_{j}$ be the vector of coefficients for all features in group *G*_*j*_. The group-lasso objective is

$$\min_{\mu ,\{\beta_{j}{\}}_{j\in 𝒞}}\frac{1}{2}\|Y-\mu 1-\sum_{j\in 𝒞}X_{j}\beta_{j}{\|}_{2}^{2}+\lambda \sum_{j\in 𝒞}\gamma_{j}\|\beta_{j}{\|}_{2}.$$ (2)

Start with $\lambda =\lambda_{\max}$, for which all coefficients are zero. Decrease *λ* to allow more terms to enter the model. Stop once a pre-specified number of interactions is selected, or choose *λ* using cross-validation or another model selection criterion.

**3. Logistic regression (binary outcome).** For a binary response Y∈{0,1}, the model is

$$\text{logit}\;P(Y=1|X)=\mu +\sum_{i=1}^{p}\left(X_{i}\theta_{i}+\sum_{j>i}X_{i:j}\theta_{i:j}\right),$$ (3)

with the same grouping structure $G_{i}=\{\theta_{i}\}\cup \{\theta_{i:j}:j>i\}$ and group-lasso penalty:

$$\min_{\mu ,\{\theta_{j}{\}}_{j\in 𝒞}}-\ell (\mu ,\{\theta_{j}\})+\lambda \sum_{j\in 𝒞}\gamma_{j}\|\theta_{j}{\|}_{2},$$ (4)

where ℓ is the log-likelihood for logistic regression.

Glinternet thus provides a robust and flexible framework for modeling interactions in high-dimensional data, ensuring interpretability through the enforcement of strong hierarchical relationships among the variables.

### Pretrained lasso

In Generalized Linear Models (GLMs) and $\ell_{1}$-regularized GLMs, an offset—a predetermined *n*-vector—can be added as an extra column in the feature matrix with a fixed weight $\beta_{j}$ of 1 that allows it to control for a variable without estimating its parameter [20]. Additionally, the standard $\ell_{1}$ norm can be extended to a weighted norm by assigning a penalty factor ${\text{pf}}_{j}\geq 0$ to each feature, modulating the regularization applied [21]. A penalty factor of zero indicates no penalty, ensuring feature inclusion, while infinity results in feature exclusion, enhancing model flexibility and specificity.

A pretrained lasso model uses both an offset and penalty factor and is fitted in two stages: first, by identifying common features, and then by isolating ancestry-specific features (Algorithm 1).

**Algorithm 1 Pretrained lasso algorithm for ancestry-diverse groups.**

Fit an overall LASSO model to the training data with all selected ancestries, selecting ${\hat{\beta }}_{0}$ along the *λ* path that minimizes cross-validation error. Set a fixed α∈[0,1]. Compute the offset and penalty factor for each input group as follows:

- Calculate the linear predictor $X_{k}{\hat{\beta }}_{0}+{\hat{\mu }}_{0}$ for each group *k*, and define the offset as $\left(1-\alpha )\cdot (X_{k}{\hat{\beta }}_{0}+{\hat{\mu }}_{0}\right)$.
- Identify the support set *S* of ${\hat{\beta }}_{0}$. For each feature *j*, define the penalty factor ${\text{pf}}_{j}$ as $\left(1-\alpha )\cdot [I(j\notin S)\cdot \frac{1}{\alpha }+I(j\in S)\right]$.

For each ancestry *k*, fit an ancestry-specific model.

- Using the defined offset and penalty factor, train an L1-penalized logistic regression model on the data of the specific ancestry.
- Use these models for group-specific predictions.

### Study population and diseases

The UK Biobank is a comprehensive cohort study that gathers data from various locations across the UK. To address the potential variability caused by population structure within our dataset, we limited our analysis to individuals who are not related, adhering to four specific criteria outlined in the UK Biobank’s sample quality control file, ukb_sqc_v2.txt. These criteria are: (1) inclusion in principal component analysis (as indicated in the used_in_pca_calculation column); (2) individuals not identified as outliers based on heterozygosity and missing data rates (found in the het_missing_outliers column); (3) absence of suspected anomalies in sex chromosome number (mentioned in the putative_sex_chromosome_aneuploidy column); and (4) having no more than ten likely third-degree relatives (as per the excess_relatives column).

We refined our study population by analyzing genetic and self-reported ancestry data, using the UK Biobank’s genotype principal components (PCs), self-reported ancestry data (UK Biobank Field ID 21000), and the in_white_British_ancestry_subset column from the sample QC file. Our focus was on individuals self-identifying as white British (337,129), non-British European (24,905), African (6,497), South Asian (7,831), and East Asian (1,704). The classification into five groups involved a two-stage process, initially using genotype PC loadings to apply specific criteria for each group: (1) white British based on PC1 and PC2 thresholds and inclusion in the white British ancestry subset; (2) non-British European with similar PC thresholds, identified as white but not white British; (3) African, with distinct PC thresholds, excluding identities as Asian, White, Mixed, or Other; (4) South Asian and (5) East Asian, each with unique PC thresholds and exclusions of identifying as Black, White, Mixed, or Other. We used both Global PCs, computed on all ancestries, and PCs, computed for a single ancestry.

We focused on ancestries representing at least 1% of the dataset, giving us a set of ancestries made of White British, Admixed (Others), Non-British European, South Asian and African. We separate White British and Non-British European as there is a PC difference between the two groups.

From this cohort, we extracted data for 96,913 individuals for which we had ancestry and demographic information, metabolites, genotype principal components (PCs), biomarkers and polygenic risk scores (PRS) from previous studies [7,8,18]. The dataset includes a wide range of variables, encompassing demographics and genetics (age, sex, PRS), lipids, cholesterol, and lipoproteins (total cholesterol, LDL, HDL, VLDL, triglycerides, phospholipids, apolipoproteins, fatty acids, and their subclasses and ratios), amino acids and metabolites (branched-chain and aromatic amino acids, glucose, lactate, pyruvate, creatinine, albumin, GlycA), lipoprotein particle sizes and subclasses (XXL, XL, L, M, S, XS for VLDL, LDL, HDL, IDL), percent compositions of lipids in different lipoproteins, and genetic principal components capturing population structure. We make the same pool of biomarkers available for each disease. The PRS were computed separately for training and testing to prevent overfitting. We use the standard PCs and Global PCs from the UK Biobank (PC 1-10 and Global PC 1-40). When training a specific model, we add its respective PRS score to the training data.

Combining these variables gave us an n×p data matrix, where n=96,913 and *p* = 303. Each row represents an observation, and each column corresponds to a variable.

Our cohort consisted of 80,810 White British (WB), 1,911 South Asians (SA), 1,499 Africans (AF), 6,783 categorized as Admixed (AD) and 5,910 Non-British Europeans(NBE). A detailed breakdown is available in Table 1. We identified 8 common diseases to use for our study for which we had a minimum of 20 positive cases per ancestry: diabetes (DIA), asthma (AST), chronic renal failure (CRF), osteoarthritis (OST), myocardial infarction (MI), cystistis (CYS), gall stones (GS) and arthritis (ART).

**Table 1:** Distribution of cases in the dataset by ancestry and disease.

Ancestry	Cases	DIA	MI	AST	GS	OST	ART	CYS	CRF
WB	Positive	5479	3234	10909	4103	7579	4969	2725	2862
WB	Negative	75331	77576	69901	76707	73231	75841	78085	77948
WB	Total	80810	80810	80810	80810	80810	80810	80810	80810
SA	Positive	460	151	285	69	101	122	56	93
SA	Negative	1451	1760	1626	1842	1810	1789	1855	1818
SA	Total	1911	1911	1911	1911	1911	1911	1911	1911
AF	Positive	232	30	201	36	86	103	24	83
AF	Negative	1267	1469	1298	1463	1413	1396	1475	1416
AF	Total	1499	1499	1499	1499	1499	1499	1499	1499
AD	Positive	632	268	932	285	492	379	234	213
AD	Negative	6151	6515	5851	6498	6291	6404	6549	6570
AD	Total	6783	6783	6783	6783	6783	6783	6783	6783
NBE	Positive	419	197	768	262	433	279	178	165
NBE	Negative	5491	5713	5142	5648	5477	5631	5732	5745
NBE	Total	5910	5910	5910	5910	5910	5910	5910	5910

### Datasets

The data set labeled “All" contains data from all ancestries available in the UK Biobank, including White British (WB) as well as ancestries South Asian, African, Admixed and Non-British European. The “Mix" dataset refers to a combination of White British data with data from one other specific ancestry at a time (e.g. White British and South Asian). This approach allows us to explore the predictive performance of our models when they are trained on data representing the majority population (White British) alongside data from a minority ancestry. The term “ancestry-specific data" is used to denote datasets consisting exclusively of data from a single, specific ancestry—such as South Asian (SA) for instance. By employing these distinct dataset configurations, our study aims to assess whether training models on all ancestries is beneficial compared to training on White British data and data from a single ancestry.

### Training and evaluation

**Fig 1:**
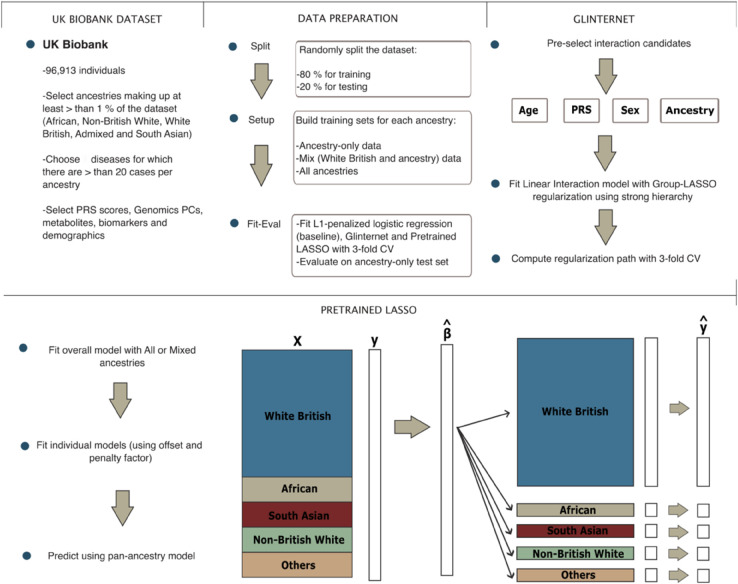
Diagram of the methodology followed for this project.

We started our analysis with a baseline model — an L1-penalized logistic regression — identified as the standard approach for this type of analysis in our literature review [22]. We used the *glmnet* package [23] in R [24] and trained it with the three distinct types of datasets aforementioned: ancestry-specific, Mix, and All (Fig 1).

Subsequently, we applied the *glinternet* package in R to train glinternet models [25], selecting age, sex, ancestry, and PRS as interaction candidates (Fig 1). Training of glinternet models is conducted on both the Mix and All datasets to assess glinternet’s ability to extrapolate patterns across ancestries facing data limitations.

Last, we adopted a two-stage process to train the pretrained lasso model. We used the *ptLasso* package in R [26] which trains a generic LASSO model on our training set using *glmnet*, from which is derived the $\hat{\beta }$ vector. This vector is then used to construct an offset parameter, facilitating the training of ancestry-specific models. A comprehensive pan-ancestry model is subsequently developed by combining these steps and is trained like the glinternet models on both the Mix and All datasets.

We divided our dataset into training and testing portions, allocating 80% for training and 20% for testing, and employed 3-fold cross-validation to identify optimal hyperparameters for our models. This cross-validation approach, using a limited number of folds, ensures the inclusion of a sufficient number of cases for each ancestry, particularly for those with restricted data.

We assessed model performance using the test portion of the dataset specific to each ancestry. We calculated the Receiver Operating Characteristic (ROC) curves and ROC-AUC scores [27] for each model and conducted DeLong tests [28] to compare the ROC curves of the glinternet and pretrained lasso models against the baseline models trained on both White British and specific ancestry data. The results include ROC-AUC scores detailed by ancestry and disease, in addition to p-values from the ROC tests and the difference in ROC-AUC (*Δ* ROC-AUC) between our models and the corresponding baseline, both trained on the same dataset. All p-values are one-sided unless specified otherwise, as we use a directional hypothesis and expect an improved performance from pretrained lasso and glinternet models compared to our baselines. We do not account for multiple testing. Statistical significance is indicated by asterisks: * for p-values less than 0.05 and ** for p-values less than 0.01.

## Results

### Results for African ancestry

For African ancestry, we find 6 statistically significant models (p-value<0.05) for 4 diseases (cystitis, gall stones, diabetes and arthritis), all of which are glinternet models. In terms of average ROC-AUC score across all diseases, pretrained lasso shows suboptimal performance and is similar or worse than the baseline models depending on the subset used for training, while glinternet slighty outperforms both the logistic regression and pretrained lasso models (Table 2).

**Table 2:** ROC-AUC scores for logistic regression, pretrained lasso and glinternet (African ancestry).

Method	Data	CRF	CYS	GL	DIA	MI	OST	AST	ART	Avg.
Logistic regression	AF	0.718	0.5	0.5	0.844	0.5	0.797	0.622	0.718	0.65
Logistic regression	Mix	0.71	0.549	0.474	0.839	0.753	0.847	0.629	0.641	0.68
Logistic regression	All	0.705	0.549	0.471	0.854	**0.755**	**0.848**	0.643	0.683	0.688
Glinternet	Mix	0.706	0.588*	0.527*	0.86*	0.754	0.84	**0.675**	0.714*	**0.708**
Glinternet	All	0.718	0.526	0.501	0.872**	0.745	0.839	0.667	0.741*	0.701
Pretrained lasso	Mix	0.689	0.568	0.478	0.858	0.63	0.788	0.639	0.697	0.669
Pretrained lasso	All	**0.725**	0.567	0.477	0.865	0.646	0.838	0.621	0.701	0.68

The glinternet approach shows significant improvements over the baseline for cystitis (*Δ* ROC-AUC of 0.039, 7.1% increase), gall stones (*Δ* ROC-AUC of 0.054, 11.2 % increase), diabetes (*Δ* ROC-AUC of 0.022, 2.5 % increase) and arthritis (*Δ* ROC-AUC of 0.073, 11.4 % increase). The performance is consistent across all diseases and is only slightly outperformed for myocardial infarction and osteoarthritis by a small margin (less than 0.01 *Δ* ROC-AUC). The glinternet models trained on the Mix datasets have an average ROC-AUC 0.028 higher than the baselines (4.1 % increase).

We find that L1-penalized Logistic Regression keeps 147 non-zero coefficients when trained on the Mix dataset for diabetes, while Pretrained LASSO keeps only 19 of them and outperforms it by a *Δ* ROC-AUC of 0.02. For Glinternet for the same disease, we report a *Δ* ROC-AUC of 0.022 and find 60 interaction terms (27 with age, 25 with the PRS for diabetes and 8 with sex).

Glinternet provides the best accuracy for predicting certain diseases in individuals of African ancestry, specifically for cystitis, gall stones, diabetes and arthritis, as shown by the ROC-AUC scores and the p-values of the ROC tests. On the contrary, pretrained lasso does not show statistically significant improvements for the ROC tests and has similar or lower average ROC-AUC scores than both baselines.

### Results for South Asian ancestry

For South Asian ancestry, we find 8 statistically significant models (p-value<0.05): five glinternet models for 3 diseases (osteoarthritis, arthritis, asthma) and 3 pretrained lasso models for 2 diseases (asthma and arthritis). Both glinternet and pretrained lasso show improved performance in terms of *Δ* ROC-AUC compared to the baselines trained on the ancestry-specific and Mix datasets, producing the top-performing ROC-AUC scores for all diseases except for cystitis and diabetes (Table 3). While the pretrained lasso models trained on the Mix dataset have similar predictive ability compared to the baselines, the best-performing approach in terms of average ROC-AUC score is pretrained lasso trained on the All dataset. The glinternet models perform similarly well in terms of average ROC-AUC, trained on either the Mix or All datasets. Asthma and osteoarthritis both have two significant *p*-values, while other disease-approach combinations show performance that is superior or on par with the baselines, with *p*-values close to the threshold for statistical significance.

**Table 3:** ROC-AUC scores for logistic regression, pretrained lasso and glinternet (South Asian Analysis).

Method	Data	CRF	CYS	GL	DIA	MI	OST	AST	ART	Avg.
Logistic regression	SA	0.748	0.5	0.5	0.782	0.675	0.706	0.5	0.706	0.639
Logistic regression	Mix	0.834	**0.663**	0.629	0.802	0.703	0.688	0.624	0.612	0.694
Logistic regression	All	0.83	0.555	0.635	**0.815**	0.714	0.691	0.639	0.646	0.691
Glinternet	Mix	0.834	0.631	**0.659**	0.814	0.718	0.736**	0.652*	0.703**	0.718
Glinternet	All	**0.84**	0.629	0.648	0.813	0.725	0.739*	0.649	0.708*	0.719
Pretrained lasso	Mix	0.829	0.63	0.551	0.807	0.746	0.714	0.642*	0.706*	0.703
Pretrained lasso	All	0.84	0.634	0.648	0.811	**0.75**	0.713	0.645	0.726*	**0.721**

Both approaches perform particularly well for arthritis, with each combination of model and dataset giving a statistically significant p-value for the ROC tests. *Δ* ROC-AUCs for those disease range from 0.062 (9.6 % increase) to 0.095 (15 % increase)

We find that L1-penalized Logistic Regression keeps 73 non-zero coefficients for arthritis, while pretrained lasso keeps only 2 of them, the PRS and sex. It outperforms it by a *Δ* ROC-AUC of 0.095. For glinternet for the same disease, we report a *Δ* ROC-AUC of 0.091 and find 60 interaction terms (27 with age, 25 with the PRS for diabetes and 8 with sex).

### Results for admixed ancestry

For Admixed ancestry, we find only 2 statistically significant models (p-value<0.05), both of which are pretrained lasso models for gall stones. We find that the Admixed glinternet and pretrained lasso models both perform better than the baselines but do not show statistically significant improvements. Pretrained lasso outperforms both baselines by a *Δ* ROC-AUC ranging from 0.036 (4.8 % increase) for both datasets. Both glinterned and pretrained lasso are the top performing approaches in terms of average ROC-AUC except for myocardial infarction and chronic renal failure (Table 4).

**Table 4:** ROC-AUC scores for logistic regression, pretrained lasso and glinternet (Admixed Analysis).

Method	Data	CRF	CYS	GL	DIA	MI	OST	AST	ART	Avg.
Logistic regression	AD	0.883	0.5	0.664	0.902	**0.816**	0.681	0.641	0.5	0.698
Logistic regression	Mix	**0.91**	0.666	0.668	0.882	0.774	0.682	0.638	0.651	0.734
Logistic regression	All	0.909	0.666	0.675	0.892	0.777	0.7	0.645	0.672	0.742
Glinternet	Mix	0.906	**0.692**	0.711	0.885	0.793	0.727	0.663	0.653	**0.754**
Glinternet	All	0.892	0.681	0.667	0.884	0.795	**0.754**	**0.673**	0.693	0.755
Pretrained lasso	Mix	0.905	0.685	0.84**	**0.948**	0.752	0.7	0.656	0.67	0.77
Pretrained lasso	All	0.906	0.65	0.866**	0.947	0.757	0.719	0.666	**0.706**	0.777

We find that L1-penalized Logistic Regression keeps 94 non-zero coefficients for gall stones, while pretrained lasso keeps only 4 of them, the PRS for gall stones and Global PCs 1, 2 and 4. It outperforms the baseline by a *Δ* ROC-AUC of 0.176. For glinternet for the same disease we report a *Δ* ROC-AUC of 0.202 and find 46 interaction terms (1 with ancestry, 10 with age, 10 with the PRS for gall stones and 10 with sex).

### Runtimes

The training times, measured in CPU time, were as follows: Glinternet models required an average of 1 hour and 48 minutes on the Mix datasets and 2 hours and 32 minutes on the All datasets. L1-penalized logistic regression models trained using glmnet took 16 minutes on the Mix datasets and 23 minutes on the All datasets. Lastly, the pretrained lasso models took 41 minutes on the Mix datasets and 1 hour and 27 minutes on the All datasets.

### Results of glinternet for arthritis for South Asian ancestry

We focus on the results of the glinternet model for arthritis trained with data from White British and South Asian ancestry to understand what is driving the prediction behind our models.

**Fig 2:**
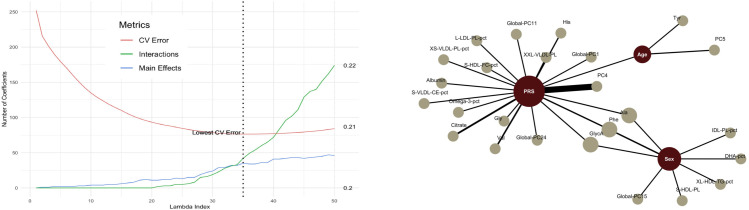
(Left) CV error vs # of main effects and interactions. (Right) Glinternet network for arthritis (SA ancestry).

The cross-validation error starts to decrease while lowering the level of regularization (Fig 2). Main effects are stable across different levels of regularization, suggesting that there are key predictors with strong associations with the outcome. As regularization is relaxed, more interaction terms are included in the model. There is a significant jump in the number of interaction terms between index 35 and 50 (from 30 to 150 interaction variables), suggesting overfitting past a certain decrease in regularization strength.

The PRS is the main covariate from which interaction terms are found. The largest interaction term found is between the PRS score and PC4 (Fig 2). It has a coefficient of 26.07 and is three times larger than the second largest interaction (variables are standardized by glinternet when fitting). In a previous study, it was found that the 4th genetic PC of the UK Biobank helped to distinguish between the different South Asian groups (Indian, Pakistani, Bangladeshi and other South Asian and mixed backgrounds) [29]. This could mean different subgroups within the South Asian ancestry have varying predispositions to arthritis.

## Discussion

We evaluated the contributions of demographic, genomic, and metabolomic variables across three ancestries (SA, AF, AD) using Logistic Regression, pretrained lasso, and glinternet models (S4 Fig). Across all ancestries, metabolomic variables consistently showed the largest coefficient weights. In Logistic Regression, metabolomic variables accounted for the majority of non-zero coefficients (averages 72–82), whereas genomic and demographic variables contributed fewer non-zero coefficients (genomic: 16–51; demographic: 1–12).

Pretrained Lasso produced sparser models, with averages of 10–23 non-zero coefficients. Despite this sparsity, metabolomic variables dominated the selected features, accounting for nearly all coefficient weight in certain diseases, such as chronic renal failure. Genomic and demographic contributions were minimal, indicating that pretrained Lasso prioritizes metabolomic signals while shrinking other variable types.

Glinternet models had a more balanced distribution of contributions. Metabolomic variables remained the largest contributors (averages 43–57), but genomic and demographic variables accounted for a notable fraction of the coefficients. Demographic variables had higher weights in Glinternet models compared with other methods, suggesting their relevance in interactions with other feature types.

These results indicate that metabolomic variables are the primary contributors across ancestries and modeling approaches. However, model choice affects the relative contributions of genomic and demographic features, with Pretrained Lasso emphasizing metabolomics and sparsity, Logistic Regression capturing a broader set of non-zero coefficients, and Glinternet balancing contributions while capturing feature interactions.

Overall, we find that the pretrained lasso and glinternet models outperform the baseline models by an average of 2 percent in ROC-AUC. The baseline method was trained and evaluated across 3 different data sets, while the glinternet and pretrained lasso methods were evaluated on 2 data sets each. Given this, the expected number of “wins” by each method under a null hypothesis of random chance is proportional to the number of data sets they were trained on. Specifically, for 24 total results (8 columns × 3 tables), the expected wins are calculated as 24×3/7=10.2 and 24× 2/7=6.9 for the glinternet method (out of 7 total methods). Observing 6 wins for the baseline and 12 wins for glinternet, a one-tailed binomial test was applied to assess whether the glinternet’s higher number of wins is statistically significant compared to chance. The test yielded a p-value of 0.022 (with alpha set at 0.025 to account for multiple comparisons), indicating marginal significance and supporting the conclusion that glinternet tends to outperform the baseline method beyond what would be expected randomly.

For 96 models trained, 16 had statistically significant incremental predictive performance in terms of ROC-AUC scores (p-value<0.05), specifically for diabetes, arthritis, gall stones, cystitis, asthma and osteoarthritis.

Pretrained lasso models were sparser than their L1-penalized logistic regression counterparts (15 coefficients on average versus 83 for logistic regression), making them easier to interpret and a superior candidate for prediction of certain diseases as they showed superior performance on average for South Asian and Admixed ancestries. They also performed better in terms of average ROC-AUC scores and p-values when trained on the All datasets versus the Mix ones. We saw no significant improvements with the pretrained lasso approach for African ancestry.

Regarding the analysis of glinternet for osteoarthritis for South Asian ancestry, adding more interaction terms between the PRS and other variables helped reach optimal performance in terms of cross-validation score, as main effects were still stable when relaxing the level of regularization. The main interaction coefficient driving the prediction was between the PRS and the 4th PC, which was used in previous studies to distinguish betweeen different ethnicities within the South Asian ancestry.

Finally, both glinternet and pretrained lasso models took a significant amount of time to train compared to the L1-penalized logistic regression baselines. This can be explained by several factors. A pretrained lasso model is made of one *glmnet* model trained on all available ancestries and *glmnet* sub-models for each ancestry used. This can make training computationally expensive, as each new ancestry added requires a separate sub-model as well as increases the size of the training set for the overall model. For glinternet, as it evaluates pairwise variable interactions for the Group-LASSO model, the complexity scales up significantly when evaluating a large number of candidate interaction terms.

### Limitations

A key limitation of this study is the use of the same dataset for both training and evaluation. Given the complexity of the models, there is a risk that they capture cohort-specific patterns that do not generalize to other populations. Future work should incorporate external datasets from additional biobanks to better assess model generalizability and to benefit from increased ancestral diversity, which may improve predictive performance.

Another limitation of our evaluation strategy is that p-values are reported only for models trained on the same dataset (e.g., Mix or All), and not across all model–training data combinations. While this enables fair comparisons under matched data conditions, it does not fully capture the clinical perspective, where one would select the single best-performing model regardless of training source. For example, some baseline models trained on the All dataset performed comparably or better than more complex approaches trained on Mix or ancestry-only data, but such comparisons are not reflected in the statistical tests. Future work could include formal pairwise comparisons across all combinations to better inform model selection in applied settings.

## Supporting information

S1 Fig Logistic regression cross-validation error over the lambda index. (PDF)

S2 Fig Glinternet cross-validation error over the lambda index. (PDF)

S3 Fig Graphs of the interaction networks of glinternet per disease and ancestry. (PDF)

S4 Fig Coefficient weights per variable type and number of non-zero coefficients for pretrained lasso, glinternet and logistic regression per ancestry. (PDF)

## Reproducibility

Our models can be accessed on Hugging Face. A permanent record of the associated materials is also available at Zenodo.
