# Supplementary figures and images for: Using pre-training and interaction modeling for ancestry-specific disease prediction using multiomics data from the UK Biobank
Source: PLoS One. 2025 Dec 1;20(12):e0336861. doi: 10.1371/journal.pone.0336861 (PMC12668563; doi:10.1371/journal.pone.0336861)

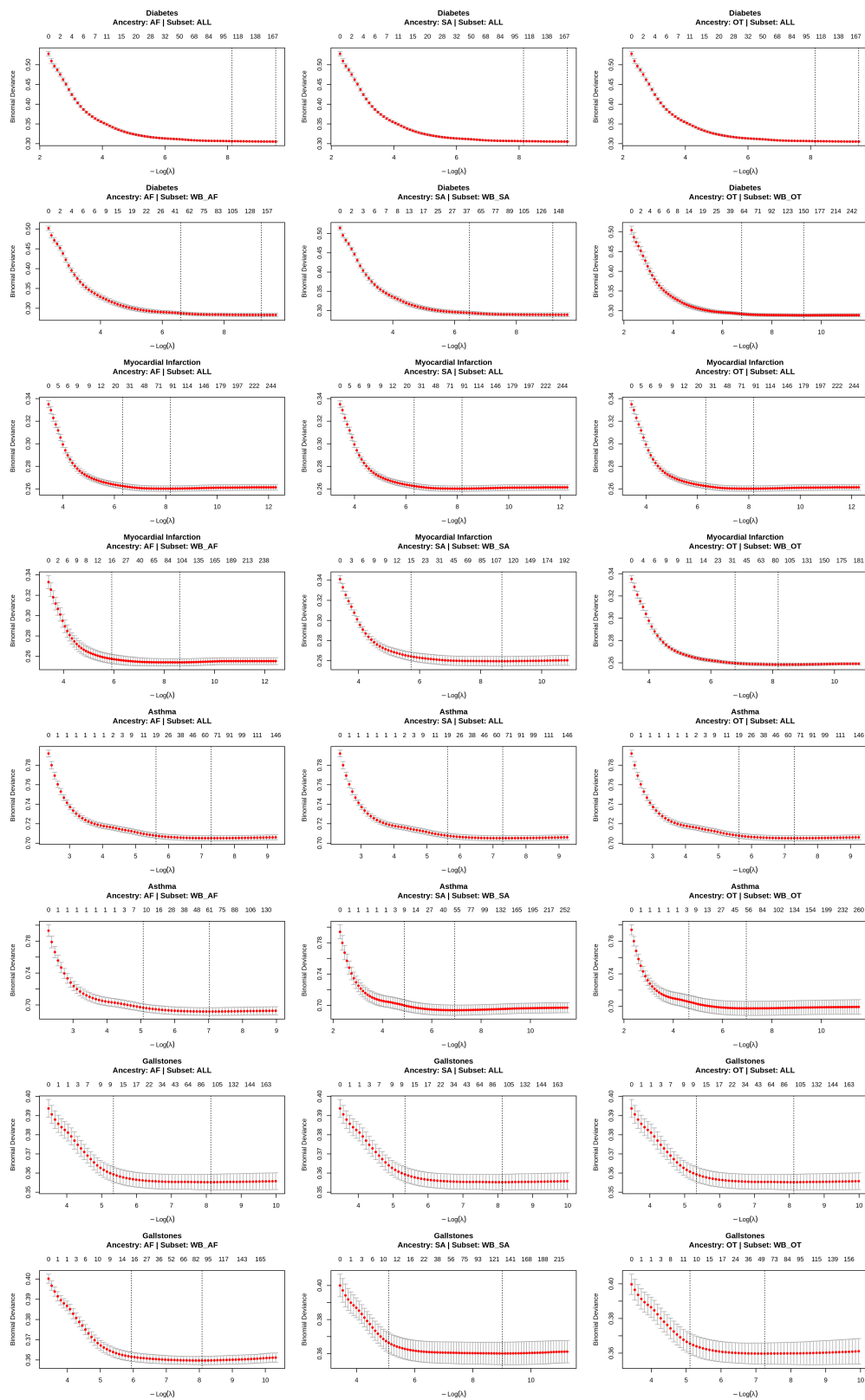

**Fig 1.** Logistic regression cross-validation error over the lambda index

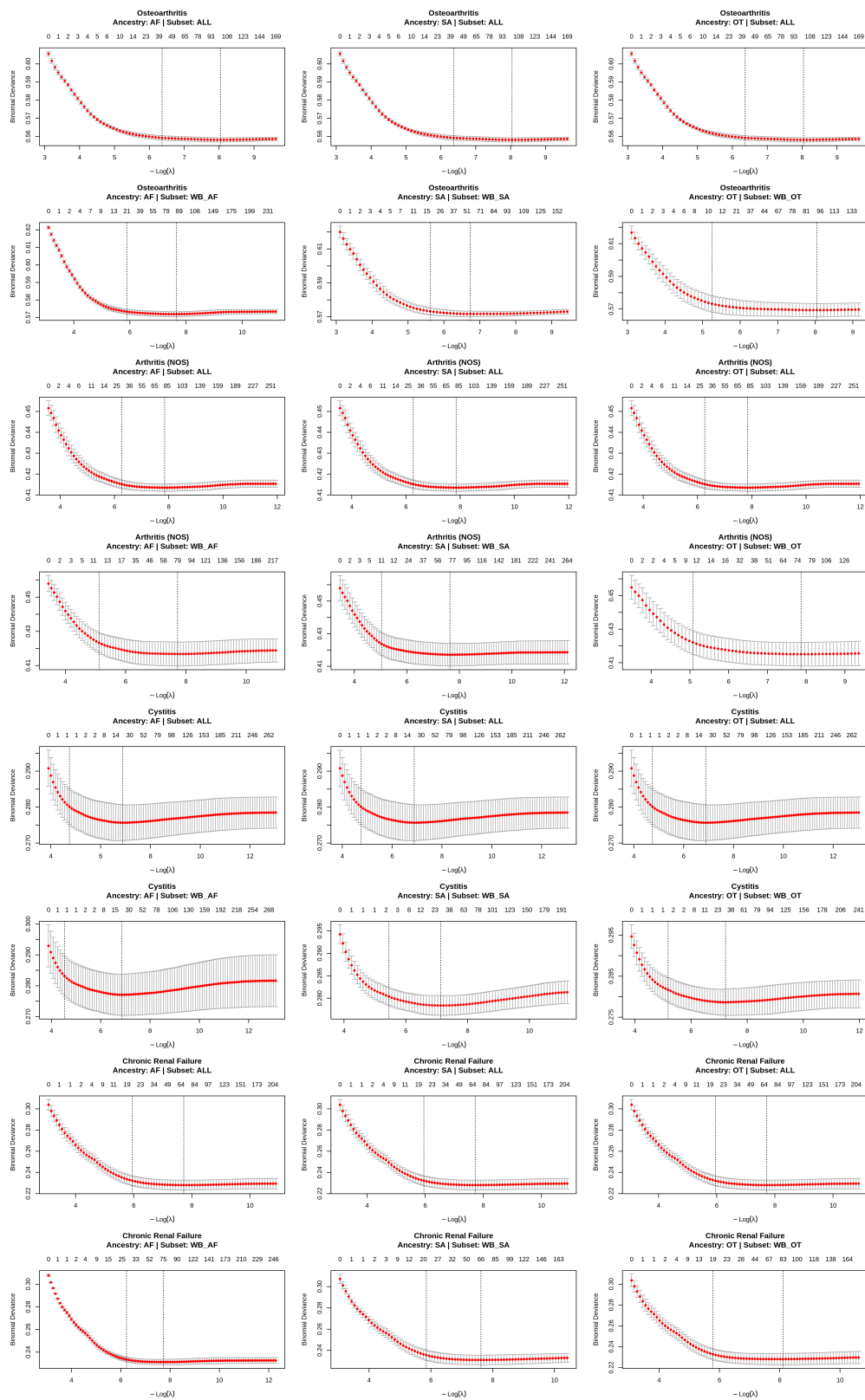

Supplement: S1 Fig — (PDF) [file pone.0336861.s001.pdf]

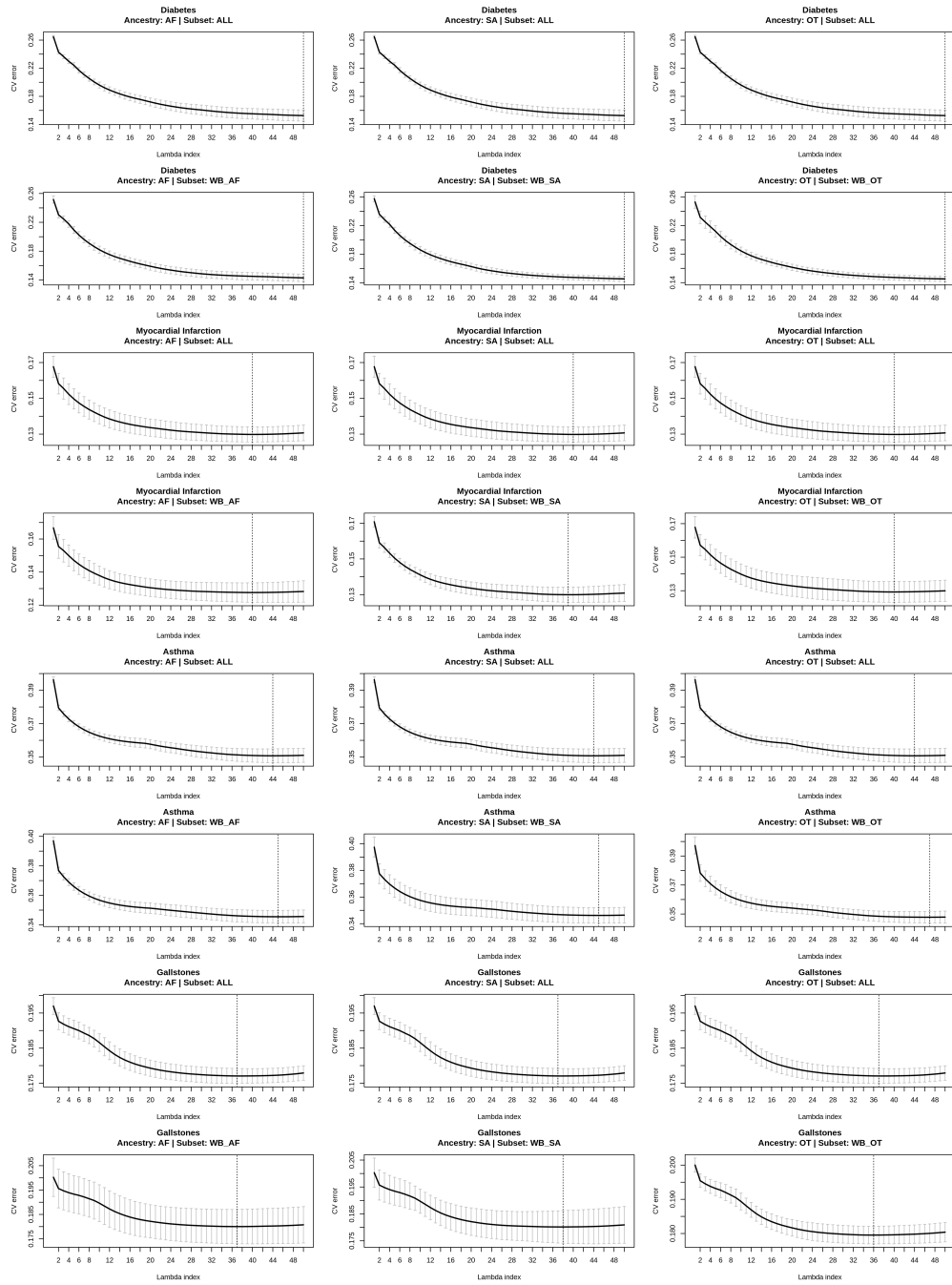

**Fig 1.** Glinetnet cross-validation error over the lambda index

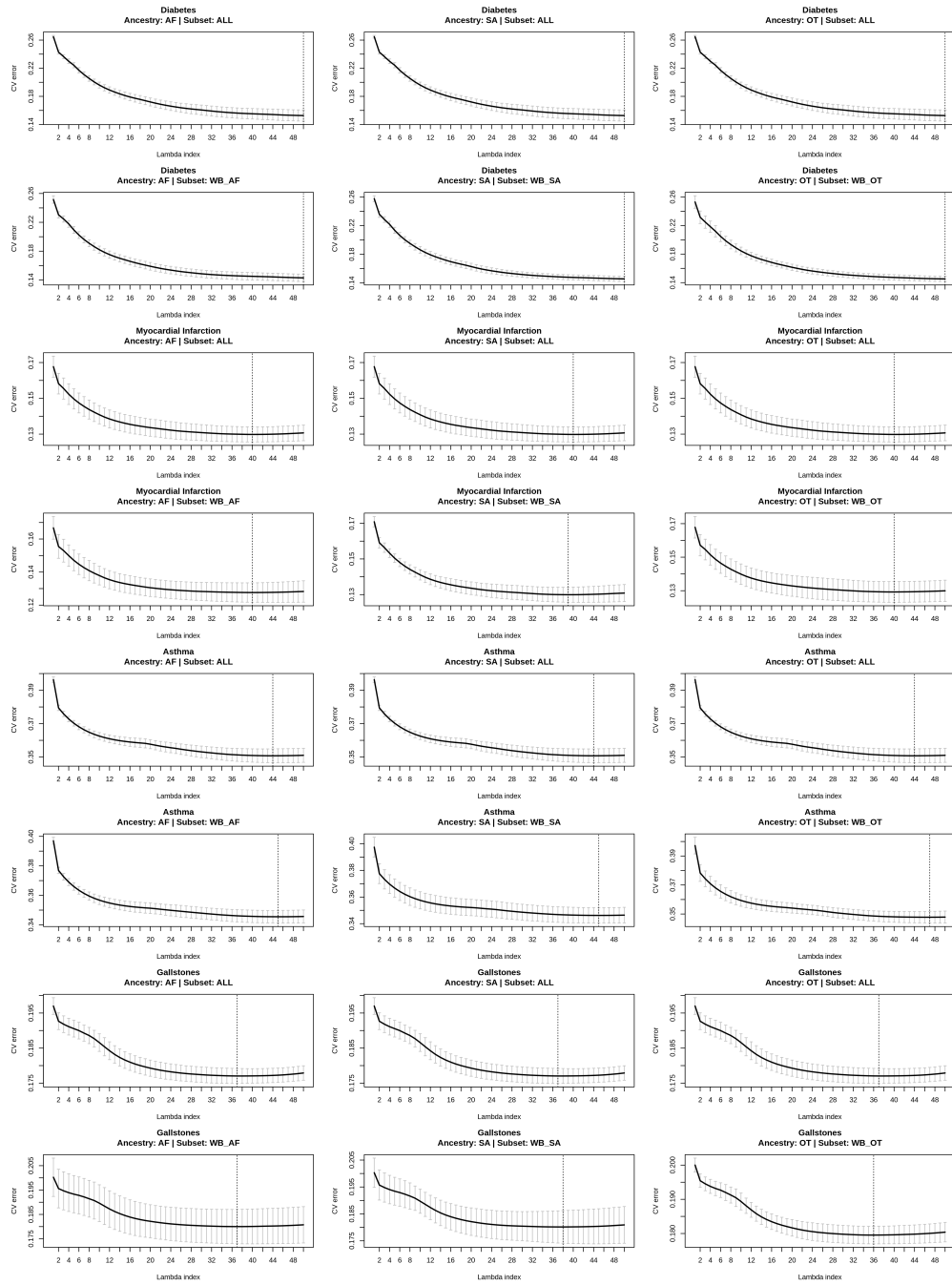

Supplement: S2 Fig — (PDF) [file pone.0336861.s002.pdf]
