# Supplementary material for: Using pre-training and interaction modeling for ancestry-specific disease prediction using multiomics data from the UK Biobank
Source: PLoS One. 2025 Dec 1;20(12):e0336861. doi: 10.1371/journal.pone.0336861 (PMC12668563; doi:10.1371/journal.pone.0336861)
Supplement: S3 Fig — (PDF) [file pone.0336861.s003.pdf]

Fig 1. Osteoarthritis glinternet interaction plots

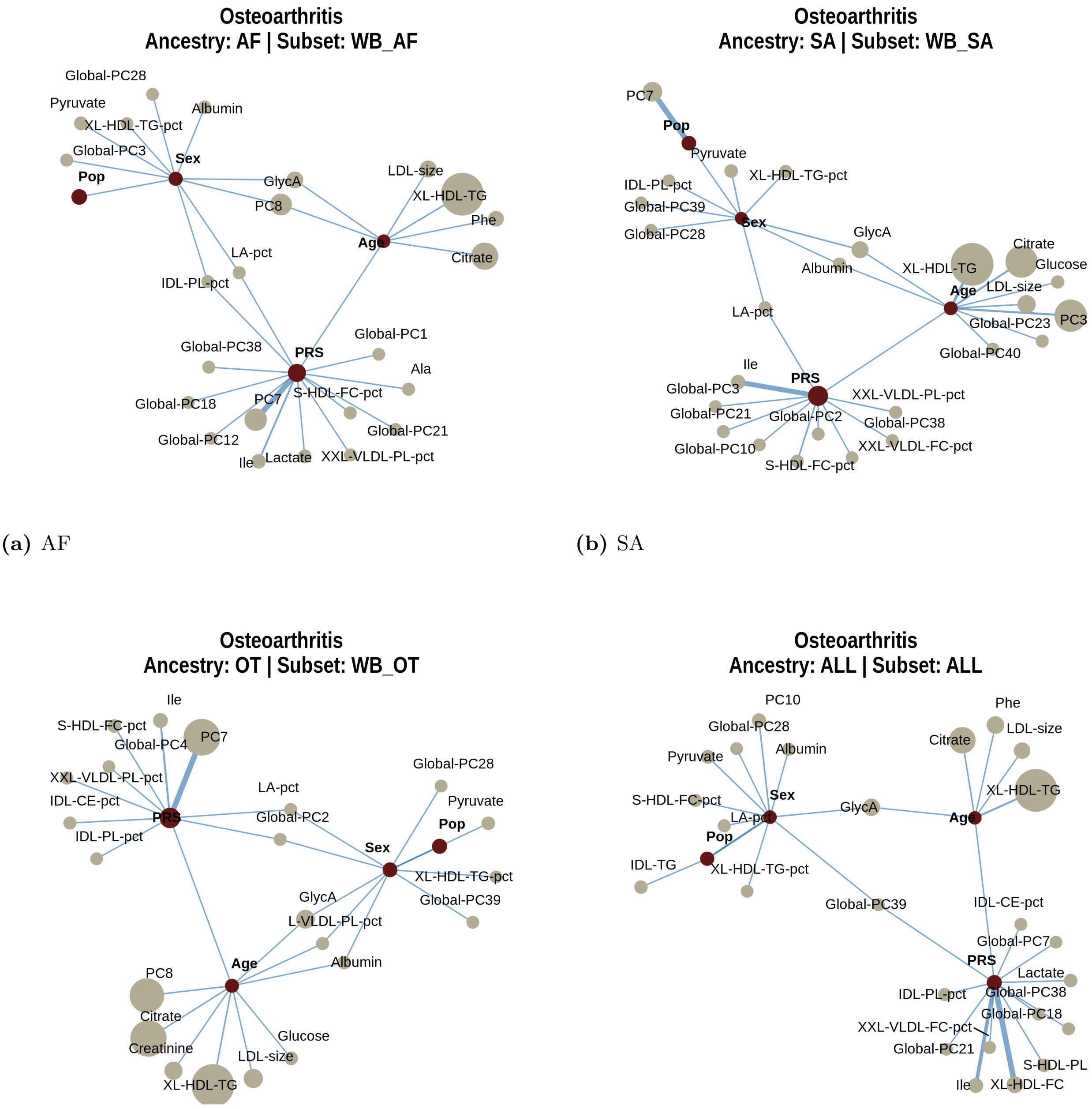

(a) AF

(b) SA

(c) OT

(d) ALL

Fig 2. Diabetes glinternet interaction plots

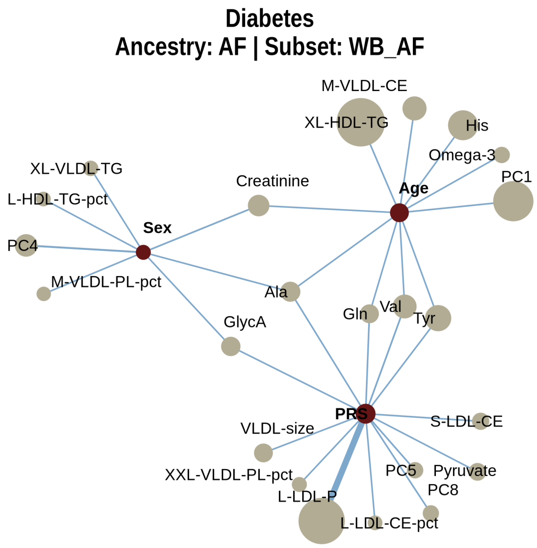

(a) AF

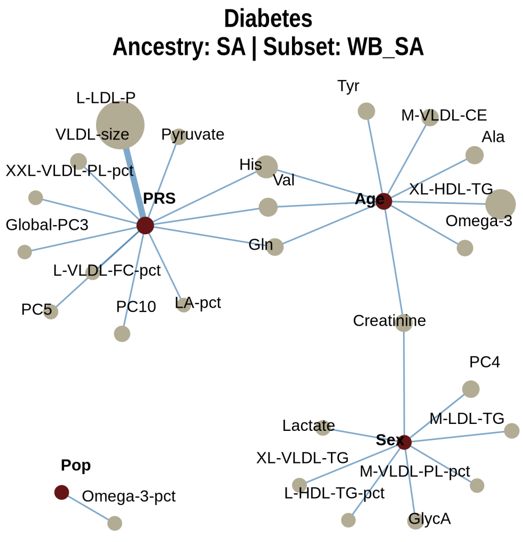

(b) SA

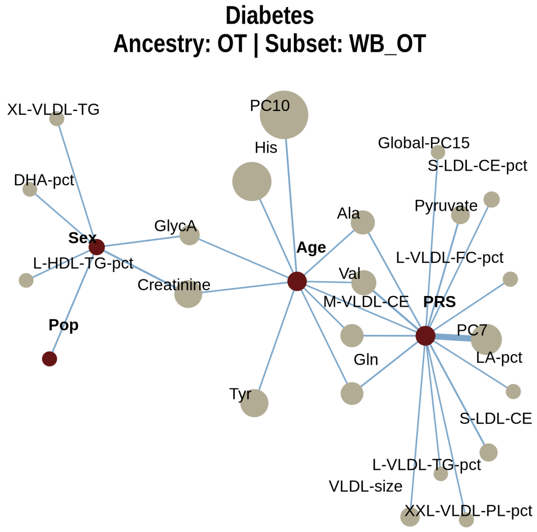

(c) OT

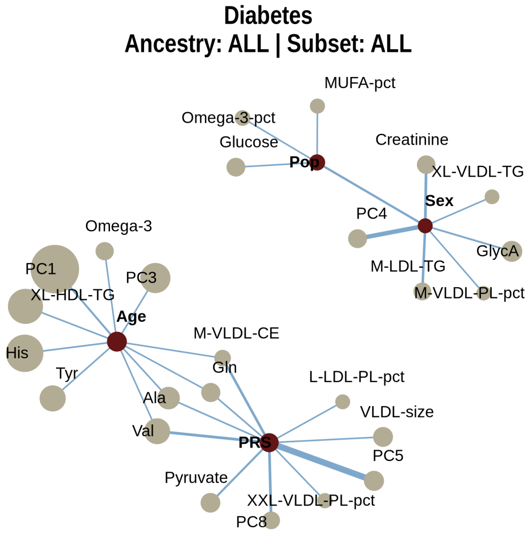

(d) ALL

Fig 3. Myocardial Infarction glinternet interaction plots

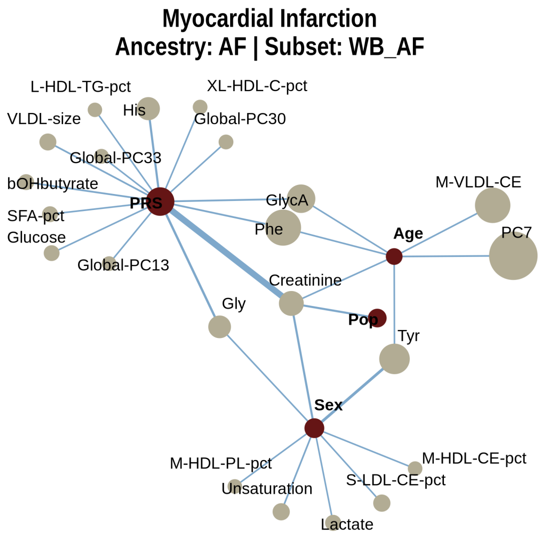

(a) AF

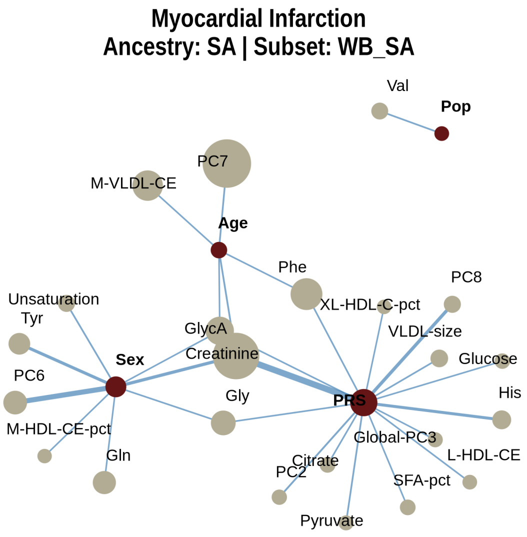

(b) SA

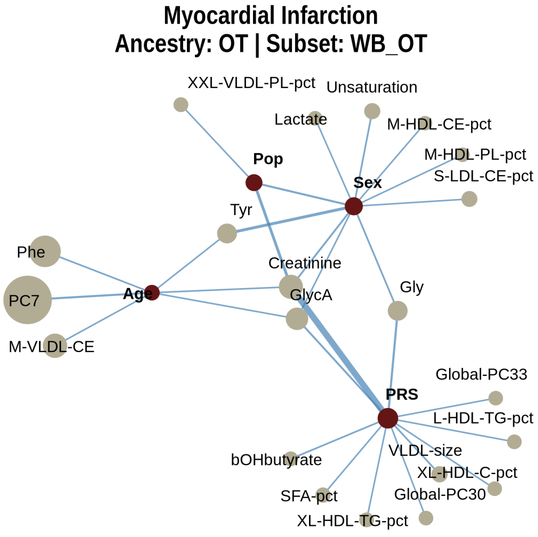

(c) OT

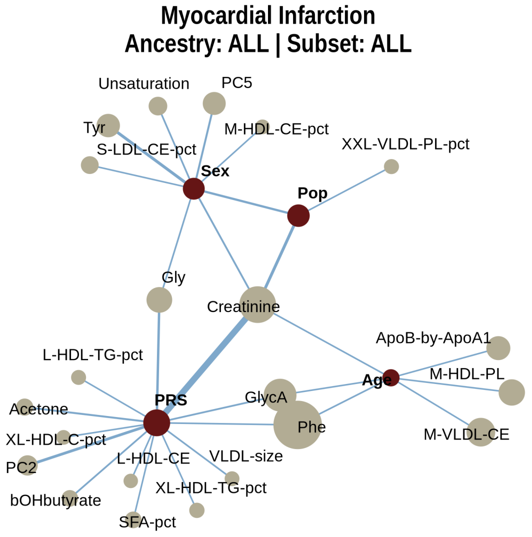

(d) ALL

Fig 4. Asthma glinternet interaction plots

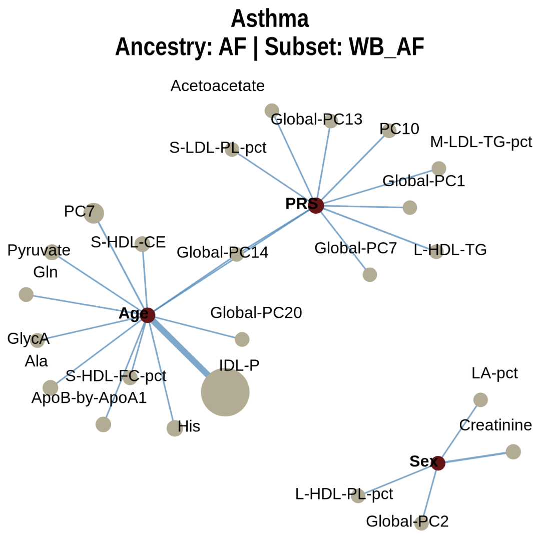

(a) AF

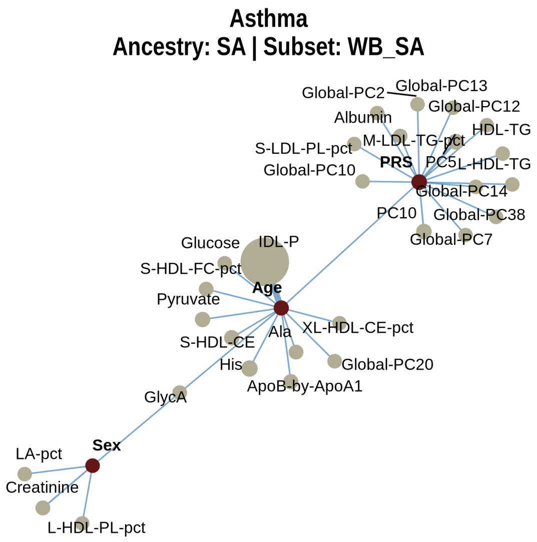

(b) SA

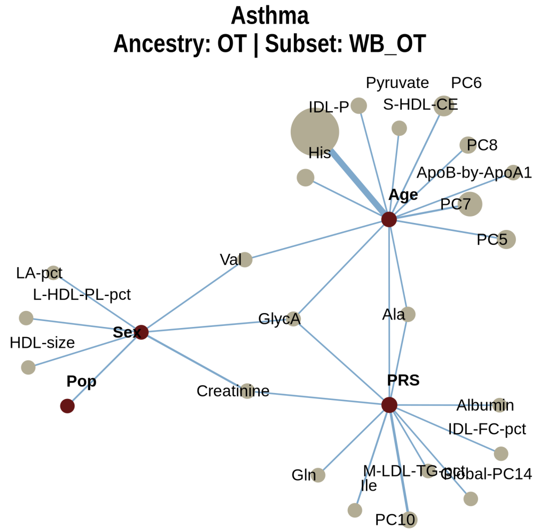

(c) OT

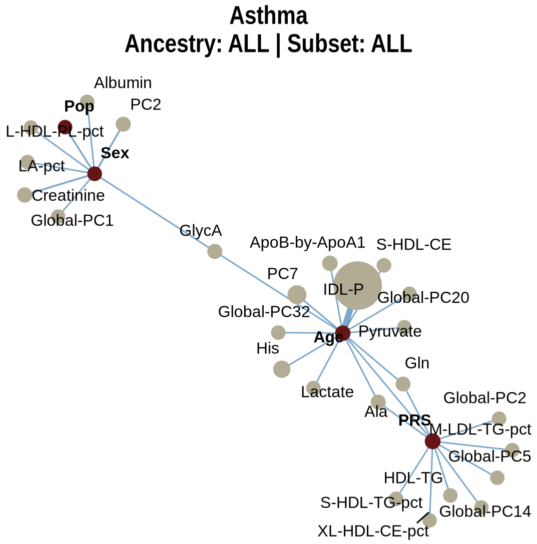

(d) ALL

Fig 5. Gall Stones glinternet interaction plots

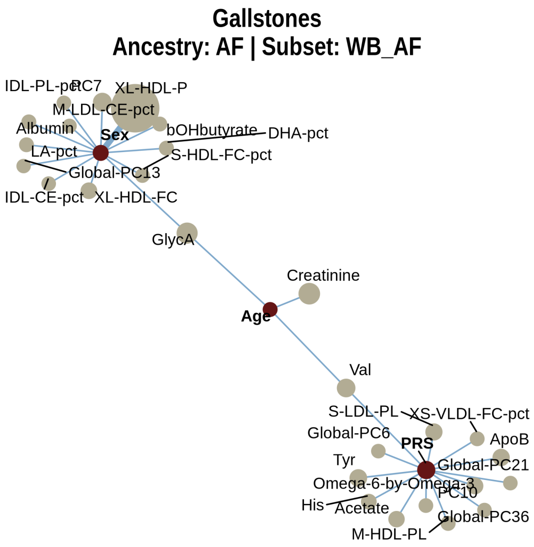

(a) AF

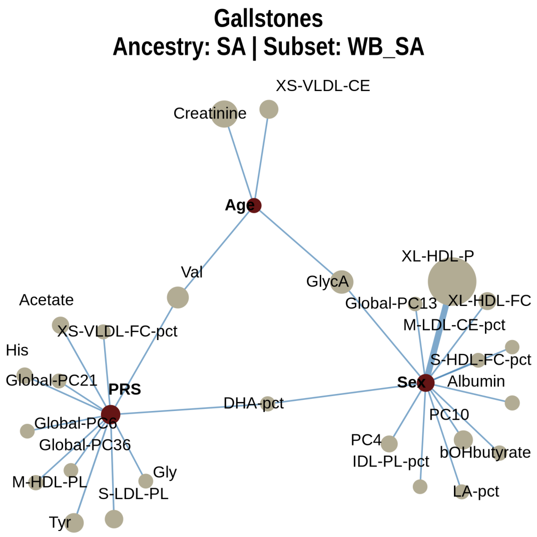

(b) SA

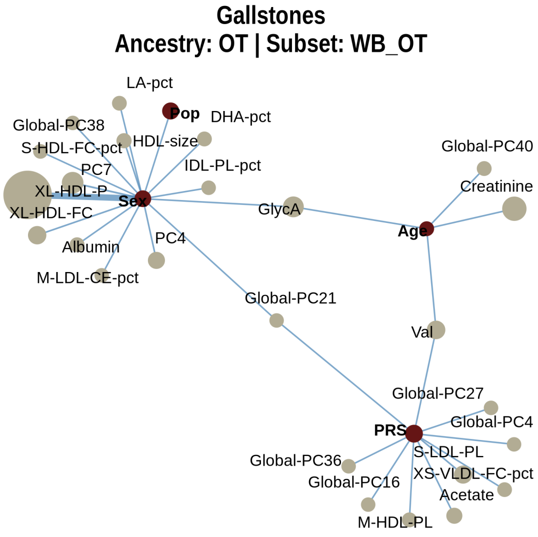

(c) OT

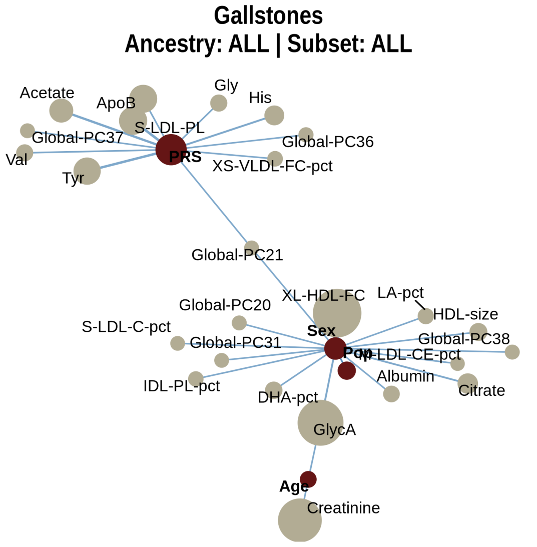

(d) ALL

Fig 6. Arthritis glinternet interaction plots

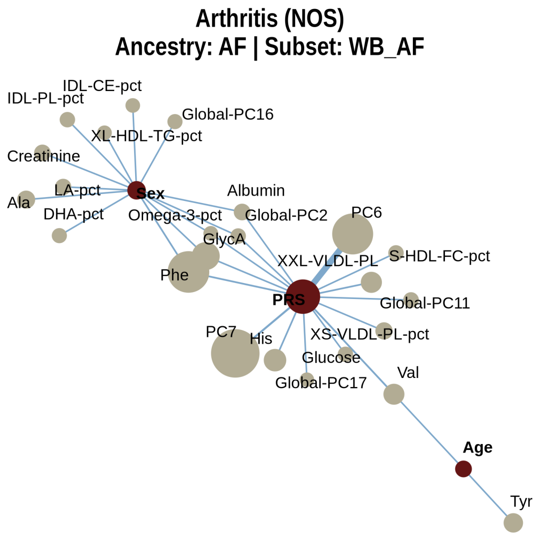

(a) AF

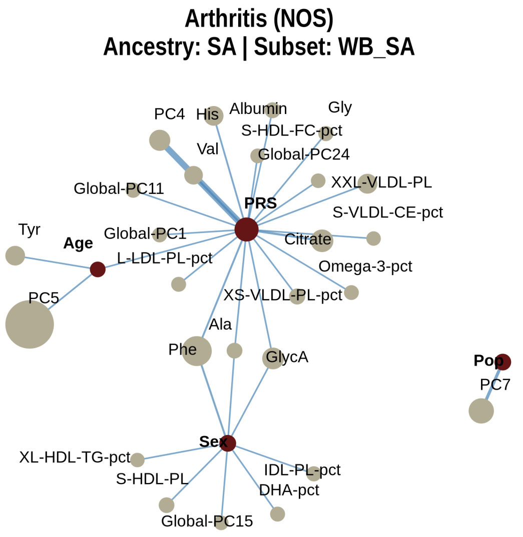

(b) SA

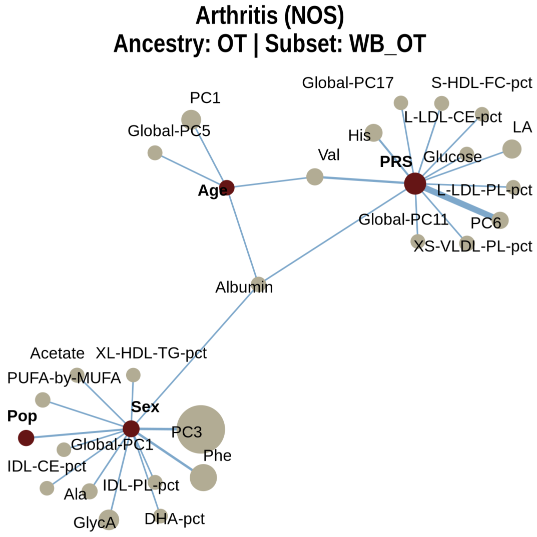

(c) OT

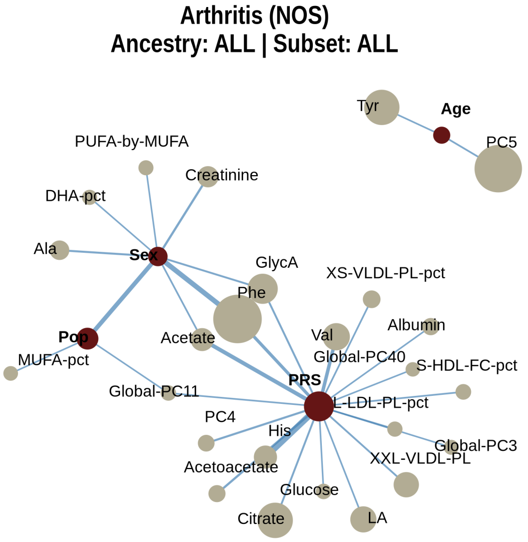

(d) ALL

Fig 7. Cystitis glinternet interaction plots

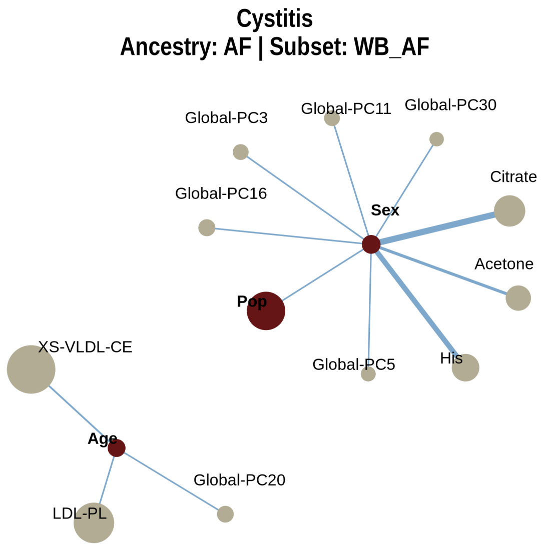

(a) AF

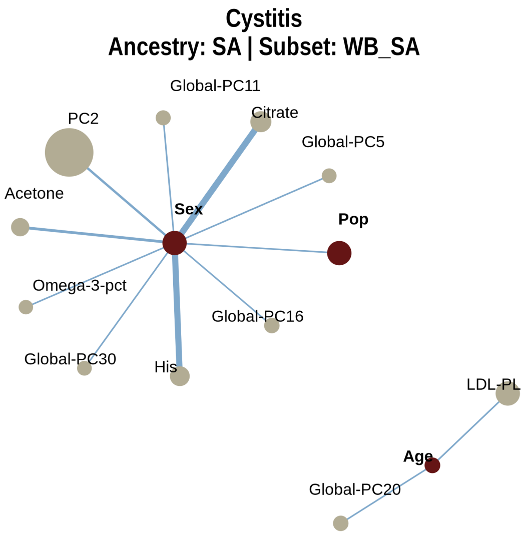

(b) SA

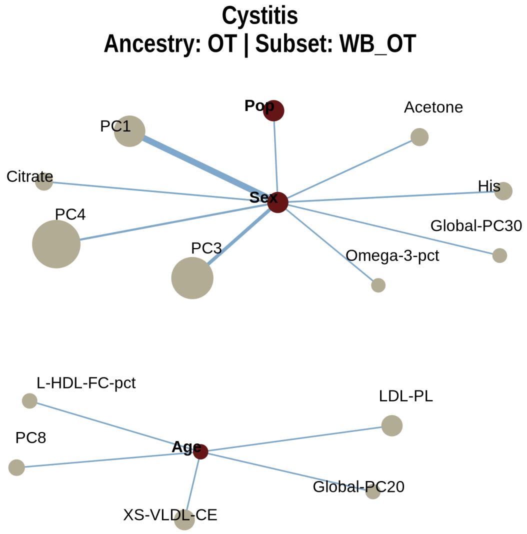

(c) OT

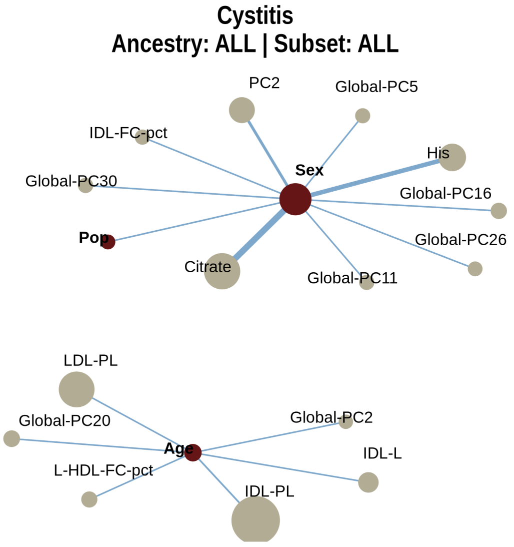

(d) ALL

Fig 8. Chronic Renal Failure glinternet interaction plots

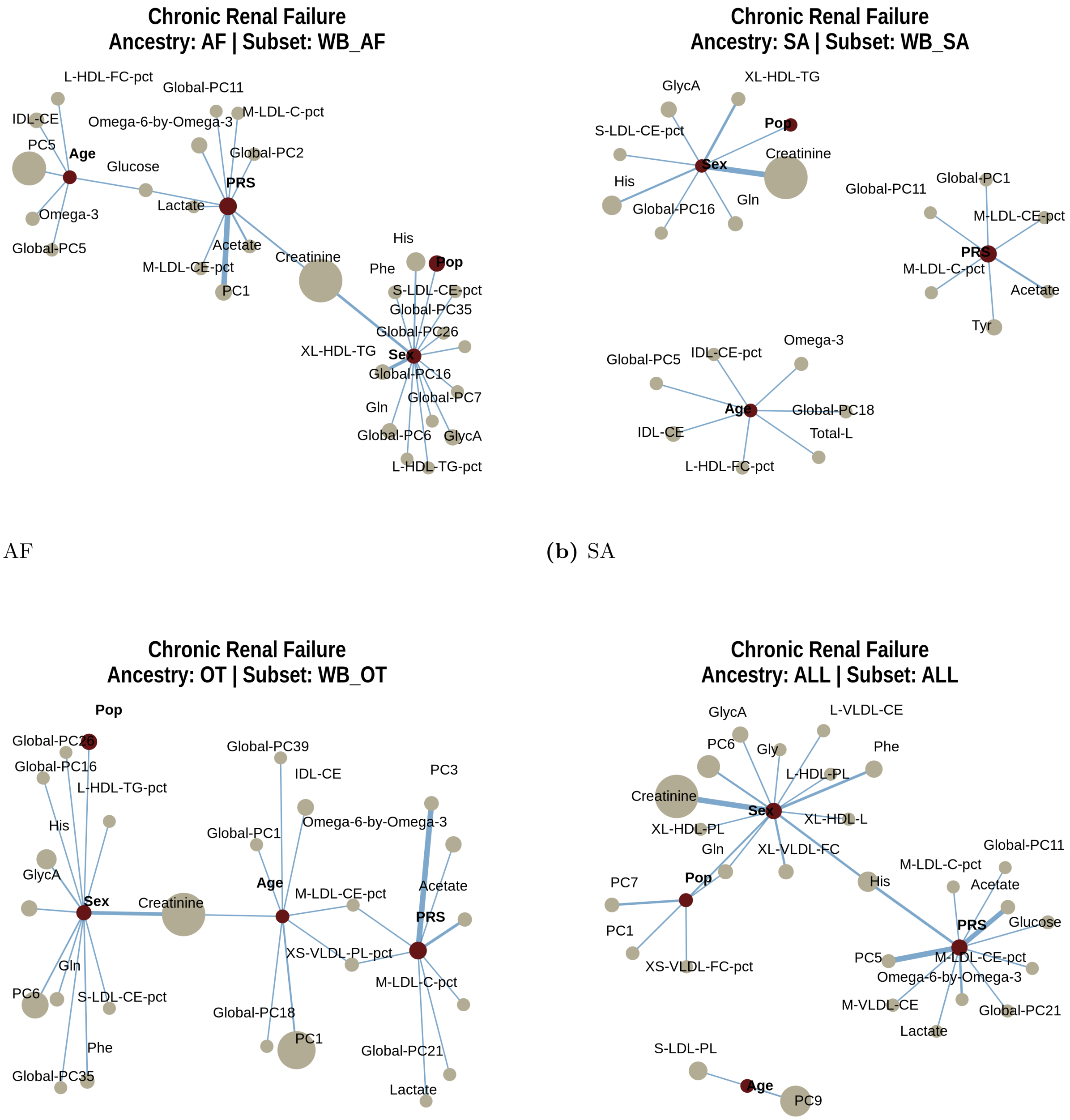

(a) AF

(b) SA

(c) OT

(d) ALL
