# Supplementary material for: Using pre-training and interaction modeling for ancestry-specific disease prediction using multiomics data from the UK Biobank
Source: PLoS One. 2025 Dec 1;20(12):e0336861. doi: 10.1371/journal.pone.0336861 (PMC12668563; doi:10.1371/journal.pone.0336861)
Supplement: S4 Fig — (PDF) [file pone.0336861.s004.pdf]

Table 1. Coefficient weights per variable type and number of non-zero coefficients for Logistic Regression (SA ancestry).

classification	CRF	CYS	GL	DIA	MI	OST	AST	ART	Average
Demographic	1	3	0	0	0	1	0	0	1
Genomic	22	79	0	7	3	62	29	16	27
Metabolomic	77	18	100	93	97	37	71	84	72
Non-zero variables	67	31	133	142	110	55	53	74	83

Table 2. Coefficient weights per variable type and number of non-zero coefficients for Pretrained lasso (SA ancestry).

Feature	CRF	CYS	GL	DIA	MI	OST	AST	ART	Average
Demographic	0	3	5	0	3	1	0	2	2
Genomic	0	85	9	0	0	75	0	10	22
Metabolomic	100	12	86	99	97	24	100	88	76
Non-zero variables	5	9	12	7	16	11	1	47	14

Table 3. Coefficient weights per variable type and number of non-zero coefficients for Glinternet (SA ancestry).

Feature	CRF	CYS	GL	DIA	MI	OST	AST	ART	Average
Demographic	6	38	27	4	8	2	1	3	12
Genomic	9	50	13	31	44	42	2	79	34
Metabolomic	84	11	59	64	46	55	96	16	54
Non-zero variables	24	14	51	61	44	30	32	37	37

Table 4. Coefficient weights per variable type and number of non-zero coefficients for Logistic Regression (AF ancestry).

Feature	CRF	CYS	GL	DIA	MI	OST	AST	ART	Average
Demographic	1	6	0	0	0	1	0	0	1
Genomic	33	68	1	3	4	55	47	7	27
Metabolomic	66	26	99	97	96	45	52	93	72
Non-zero variables	76	27	84	148	103	86	62	80	83

Table 5. Coefficient weights per variable type and number of non-zero coefficients for Pretrained lasso (AF ancestry).

Feature	CRF	CYS	GL	DIA	MI	OST	AST	ART	Average
Demographic	0	2	0	0	0	4	6	6	2
Genomic	0	81	3	2	2	6	2	0	12
Metabolomic	100	17	97	98	98	89	91	94	86
Non-zero variables	4	18	10	10	114	16	6	3	23

Table 6. Coefficient weights per variable type and number of non-zero coefficients for Glinternet (AF ancestry).

Feature	CRF	CYS	GL	DIA	MI	OST	AST	ART	Average
Demographic	6	30	25	8	5	2	2	2	10
Genomic	30	0	10	33	50	46	5	83	33
Metabolomic	62	69	64	57	44	50	92	14	57
Non-zero variables	34	14	47	49	37	41	27	31	35

Table 7. Coefficient weights per variable type and number of non-zero coefficients for Logistic Regression (OT ancestry).

Feature	CRF	CYS	GL	DIA	MI	OST	AST	ART	Average
Demographic	0	1	3	0	1	0	0	0	1
Genomic	52	89	41	15	44	65	31	74	51
Metabolomic	48	10	56	85	55	35	69	26	48
Non-zero variables	84	28	52	151	87	95	55	77	79

Table 8. Coefficient weights per variable type and number of non-zero coefficients for Pretrained lasso (OT ancestry).

Feature	CRF	CYS	GL	DIA	MI	OST	AST	ART	Average
Demographic	0	1	0	0	0	0	0	0	0
Genomic	0	89	3	2	13	13	0	0	15
Metabolomic	100	11	97	98	86	86	100	100	85
Non-zero variables	2	28	2	27	9	5	2	1	10

Table 9. Coefficient weights per variable type and number of non-zero coefficients for Glinternet (OT ancestry).

Feature	CRF	CYS	GL	DIA	MI	OST	AST	ART	Average
Demographic	3	26	28	11	10	2	2	5	11
Genomic	36	69	6	48	51	54	15	82	46
Metabolomic	60	4	65	40	38	42	81	12	43
Non-zero variables	28	16	32	46	38	27	45	29	33

Table 10. Coefficient weights per variable type and number of non-zero coefficients for Logistic Regression (ALL ancestry).

Feature	CRF	CYS	GL	DIA	MI	OST	AST	ART	Average
Demographic	1	12	0	0	1	1	0	0	2
Genomic	14	18	0	0	35	37	17	4	16
Metabolomic	85	70	100	100	64	62	82	96	82
Non-zero variables	70	23	100	175	84	105	67	81	88

Table 11. Coefficient weights per variable type and number of non-zero coefficients for Pretrained lasso (ALL ancestry).

Feature	CRF	CYS	GL	DIA	MI	OST	AST	ART	Average
Demographic	0	1	8	0	3	2	0	5	2
Genomic	0	86	13	1	1	9	4	10	16
Metabolomic	100	13	79	99	97	89	96	86	82
Non-zero variables	2	22	12	26	10	6	12	8	12

Table 12. Coefficient weights per variable type and number of non-zero coefficients for Glinternet (ALL ancestry).

Feature	CRF	CYS	GL	DIA	MI	OST	AST	ART	Average
Demographic	11	55	17	18	14	3	2	8	17
Genomic	33	3	35	44	29	10	3	50	26
Metabolomic	54	41	46	37	55	85	94	40	57
Non-zero variables	52	18	41	52	43	38	32	38	39
